# Too many too soon - fever following PET CT

**DOI:** 10.1186/2047-2994-4-S1-P256

**Published:** 2015-06-16

**Authors:** A Ghafur, PR Vidyalakshmi, K Mageshkumar, C Karupusamy, I Poojary

**Affiliations:** 1 Infection Control, Apollo Speciality Hospitals, Chennai, India; 2 Infectious Diseases, Apollo Speciality Hospitals, Chennai, India; 3 Microbiology, Apollo Speciality Hospitals, Chennai, India

## Introduction

Contaminated medical equipment and intravenous fluids are well known causative factors for procedure related bacteremia.

## Objectives

Outbreak investigation we carried out in May 2014 in our PET CT unit.

## Methods

Four of our patients who had PET CT in May 2014 presented with acute onset febrile illness shortly after the PET. All patients who had PET CT at that time were followed up. Three out of 4 patients grew *Serratia* in blood. Culture of medication samples including contrast and Saline bottles along with environmental samples and hands of staff were carried out.

## Results

Four patients had fever following PET CT procedure in May 014. All four patients had a PET CT as a part of evaluation of malignancy. Three out of four had *Serratia* marsescens bacteremia with the same antibiogram.

Investigation for the possible source revealed that a normal saline bag (multi dose vial) that was used to reconstitute the contrast was the point source for the outbreak. Sample from the bag grew *Serratia* with the same antibiogram as that of the bacteremic patients. Although analysis by pulsed-field gel electrophoresis (PFGE) was not carried out, there was no other obvious source of contamination and the patients who developed bacteremia had no other intervention other than the PET scan. Post investigation corrective action was termination of the practice of using multi dose normal-saline bag and replacing those with single use saline flush for each patient. There were no further cases of *Serratia* bacteremia in PET CT unit since then. All the three patients recovered on antibiotic treatment.

## Conclusion

It is extremely important to be vigilant to detect outbreaks at the outset itself, especially in units where investigations are carried out in an out patient basis. The study also underscores the importance using single dose vials/single saline flush for all procedures including radiology ones.

## Disclosure of interest

None declared.

